# Insights of undergraduate health sciences students about a French interprofessional training initiative

**DOI:** 10.1186/s12909-024-05212-9

**Published:** 2024-03-01

**Authors:** Mélanie Gaillet, Patrice François, Guillaume Fond, Rebecca Shankland, Maria de Fatima Novais, Julien Provost, Marie Herr, Laurent Boyer, Bastien Boussat

**Affiliations:** 1 Department of Clinical Epidemiology, Grenoble-Alps University Hospital, Laboratory TIMC-IMAG, UMR 5525 Joint Research Unit, National Center for Scientific Research, Faculty of Medicine, Grenoble Alps University, Grenoble, France; 2 School of Medicine - La Timone Medical Campus, AP-HM, Aix-Marseille University, UR3279: Health Service Research and Quality of Life Center (CEReSS), Marseille, France; 3 Laboratoire DIPHE, Université Lumière Lyon 2Institut Universitaire de France, Paris, Lyon, France; 4 Department of Nursing Education, Centre Hospitalier Alpes Isère, Saint-Egrève, France; 5 Department of Physiotherapy, Grenoble-Alps University, Grenoble, France; 6 Inserm, Anti-Infective Evasion and Pharmacoepidemiology Epidemiology and Public Health Department, AP-HP, UVSQ, University of Paris-Saclay, University of Paris-Saclay, Montigny Le Bretonneux, Paris, France; 7 O’Brien Institute for Public Health, University of Calgary, Calgary, AB Canada

**Keywords:** Interprofessional education, Undergraduate students, Primary prevention, Health promotion, Public health service

## Abstract

**Background:**

Incorporating interprofessional collaboration within healthcare is critical to delivery of patient-centered care. Interprofessional Education (IPE) programs are key to promoting such collaboration. The ‘Public Health Service' (PHS) in France is a mandatory IPE initiative that embodies this collaborative spirit, bringing together students from varied health undergraduate training programs—nursing, physiotherapy, pharmacy, midwifery, and medicine— in a common training program focused on primary prevention. The aim of the study was to assess the experience and attitudes of students in the five health training programs regarding the interest of IPEs in the PHS.

**Methods:**

A cross-sectional survey was administered to 823 students from the 2022–2023 cohort at a French university. The questionnaire was designed with 12 Likert-scale questions specifically created to evaluate the students' experiences, knowledge, and attitudes focused on IPE during the practical seminars, school interventions, and the overall PHS. Additionally, an open-ended question was utilized to gather qualitative data. Statistical analyses assessed satisfaction levels across undergraduate training programs, while thematic analysis was applied to the qualitative responses.

**Results:**

Within the surveyed cohort, 344 students responded to the survey. The findings showed that students were satisfied with the interprofessional collaboration, both in practical teaching sessions (75% satisfaction) and in primary prevention projects conducted in schools (70% satisfaction), despite their having faced challenges with coordination. Pharmacy students, in particular, highlighted the need for adjustments in program scheduling. The qualitative feedback underscored the positive value of IPE, notwithstanding the organizational difficulties stemming from different academic timetables.

**Conclusion:**

The student feedback indicated a high level of satisfaction with the interprofessional work carried out in both the practical teaching and the primary prevention projects. To further enhance the educational impact and address the scheduling complexities, it is recommended that program refinements be made based on student feedback and pedagogical best practices.

**Supplementary Information:**

The online version contains supplementary material available at 10.1186/s12909-024-05212-9.

## Introduction

Interprofessional collaboration in healthcare has increasingly been recognized as an essential component in delivery of comprehensive and patient-centered care [1–3]. By amalgamating varied professional perspectives, interdisciplinary approaches ensure a holistic understanding of health and illness, bridging gaps that may exist when disciplines work in silos. For instance, Interprofessional Education (IPE) programs bring together students from diverse health professions to learn from, with, and about each other, fostering collaboration and mutual understanding [4–6].

To harness the full potential of interdisciplinary collaboration, numerous initiatives have been introduced globally, targeting improvement in teamwork, communication, and shared decision-making [7–9]. These initiatives not only foster professional respect and trust, but also help to optimize patient outcomes and to ensure efficient resource utilization. Morbidity and Mortality Review Boards, for example, provide a platform for healthcare professionals to collaboratively analyze and learn from clinical errors and adverse events [10]. This collective approach to problem-solving enriches the learning experience and enhances the quality of patient care.

Despite its evident importance, interdisciplinary training remains underrepresented in the initial educational stages of many health professions [11]. A gap exists between the recognized need for such collaboration in clinical settings and the preparation students receive during their formative years. This discrepancy underscores the need for innovative educational strategies that embed interdisciplinarity early in professional development, as exemplified by the IPE programs [6].

Enhancing interdisciplinary collaboration is a principal aim of the ‘Public Health Service’ (PHS, i.e. known as “Service Sanitaire” in French), a mandatory program introduced by the French government into the healthcare students' curriculum in 2018 [12, 13]. This program is centered on primary prevention, providing medical, pharmacy, midwifery, physiotherapy, and nursing students with foundational training in health promotion concepts and techniques [14]. As part of this program, students from the University of Grenoble-Alpes (UGA) are required to collaboratively design and implement a health education project aimed at schoolchildren [15, 16]. By focusing on primary prevention and facilitating hands-on interventions in educational institutions, from kindergartens through high schools, the program serves as a practical platform for the fostering of interprofessional collaboration and understanding [17, 18].

This study endeavors to shed light on the perceptions of students enrolled in the PHS program of UGA. Specifically, it aims to explore their viewpoints on the benefits and challenges of interdisciplinary training and to develop insights on the program's potential role in shaping future healthcare professionals equipped for collaborative practice. Through this exploration, the study hopes to contribute to the ongoing global dialogue on the importance of interdisciplinary education in healthcare.

## Methods

### Study design

This was an online questionnaire survey conducted among students who participated in the PHS program at the UGA during the 2022–2023 academic year.

### Context

The UGA PHS, established in 2018, includes students from five undergraduate training programs, with their inclusion in their respective years of study—medicine in the 3rd year, nursing in the 2nd year, physiotherapy in the 3rd year, midwifery in the 2nd year, and pharmacy in the 5th year— having been set by governmental decree. These students participate in a unified training program involving health education initiatives in schools, which is coordinated in partnership with the educational authorities of our geographical region's academy. Prior to the school year, a survey is conducted by the academy's administrative authorities to identify schools willing to participate in the program. This results in a list of schools ready to host PHS students for primary prevention interventions. Detailed descriptions of the program's structure and its comprehensive approach have been published elsewhere [18]. In summary, the program includes:

-A Theoretical e-learning training with audio lectures, documents, and references.
- Practical training through two-day seminars for groups of 16 to 20 students led by two teachers from the undergraduate training programs involved. The seminar focuses on the acquisition of educational competencies and the development of psychosocial skills.
- Health education intervention in schools. Having expressed their geographical preferences, the students are divided among volunteering schools, in groups of 2 to 12. Each group must contact the school principal, analyze the context and the request (themes and classes), and agree with him or her on the content and schedule of the intervention. The intervention plan, which must include five one-hour health education sessions per class, is submitted for validation to a teacher, who is the pedagogical referent of the student group. Each student group carries out the intervention according to the validated plan.

The evaluation and validation of the PHS include the submission of an intervention report by each student group, validation of the report by the group's pedagogical referent, an assessment from the school principal, and a knowledge check through an individual exam.

### Unified academic schedule for the five health undergraduate training programs

To facilitate an integrated approach within the PHS, a harmonized academic calendar has been established for students enrolled in the five health-related training programs. The calendar designates Monday afternoons as a dedicated time for students to engage collaboratively in the PHS activities, extending over a period of three months. During this time, students are expected to jointly develop their intervention strategies and execute a series of five health education sessions in local schools, utilizing the allocated Monday afternoon time slots.

### Study population

The online questionnaire was sent to all 823 students who completed the UGA PHS in the 2022–2023 academic year, including 354 second-year nursing students, 287 third-year medical students, 83 fifth-year pharmacy students, 65 fourth-year physiotherapy students, and 34 second-year midwifery students.

### The questionnaire

The questionnaire was designed with 12 Likert-scale questions specifically created to evaluate the students' experiences, knowledge, and attitudes toward IPE during the practical seminars, school interventions, and the overall PHS. The Likert scale provided a range from 'Strongly Disagree,' 'Disagree,' 'Neutral,' 'Agree,' to 'Strongly Agree' to capture the nuances of the students' responses. Additionally, the questionnaire included one open-ended question for free-form comments to allow for in-depth qualitative feedback.

The responses were anonymous; students were only required to indicate their undergraduate training program and the category of the school where they intervened.

### Data collection

The questionnaire was distributed via the Sphinx application in June 2023, three months after completion of the PHS, with participants being reminded up to three times. Survey data were supplemented with information from PHS management documents: intervention reports and the composition of student groups during seminars.

### Analysis

#### Statistical analysis

For closed questions, the responses were dichotomized, and we reported the percentage of positive responses ("agree" and "strongly agree"), excluding other responses. The percentage of positive responses was calculated for each question and each undergraduate training program. Associations between variables were analyzed using the chi-squared test. Quantitative variables were expressed as medians and interquartile ranges, and associations were analyzed using the Kruskal–Wallis test. Analyses were performed with Stata SE software (version 16, StataCorp, College Station, TX, USA). The significance threshold for the tests was 0.05.

### Analysis of comments

Comments were collected in the comments section of the questionnaire. We initially isolated all open-ended comments to form a comprehensive database. A preliminary screening for IPE-related comments was conducted through a general reading by two investigators (BB & PF), which led to the identification of a subset of comments specifically related to the theme of IPE. These IPE-related comments were then subjected to an independent thematic analysis conducted by the same investigators. During this process, each comment was categorized into the relevant topic, and in instances where comments spanned multiple topics, they were correspondingly tagged with multiple occurrences across different topics. Additionally, keywords were assigned to each comment to succinctly capture its essence. To ensure objectivity and consistency in the thematic analysis, the investigators compared their findings and any discrepancies were reconciled by a third investigator (LB).

## Results

### Study population

Out of the 823 students participating in the 2022–2023 PHS and targeted for the survey, 344 (42%) responded to the questionnaire. This included 156/354 nursing students (44%), 106/287 medical students (37%), 26/83 pharmacy students (31%), 31/65 physiotherapy students (48%), and 25/34 midwifery students (74%). These students carried out their health education interventions in 113 schools, encompassing 24 kindergartens, 73 primary schools, 7 middle schools, and 9 high schools (Table 1). Their interventions, consisting of 5 sessions of 1 h each, engaged 8,061 pupils. Based on the intervention reports, the most frequently addressed health education topics were screen usage (in 39% of the schools), nutrition (37%), personal hygiene (26%), domestic risks (22%), psychosocial skills (20%), physical activity (14%), and bullying (13%). Analysis of student’s intervention reports is shown in the Supplementary material 1.

**Table 1:** General characteristics of institutions, students, and undergraduate training program

	Kindergarten		Elementary School		Middle School		High School		Total
	n	%	n	%	n	%	n	%	n
Number of institutions (n, %)	24	21%	73	65%	7	6%	9	8%	113
Number of pupils (n, %)	1399	17%	5730	71%	453	6%	479	6%	8061
Pupils per institution (median, [IQR])	54 [40; 75]		77 [50; 100]		60 [50; 90]		40 [27; 75]		60 [48; 97]
Number of students (n, %)	143	17%	582	71%	36	4%	62	8%	823
- Nursing care (n, %)	50	14%	74	21%	22	6%	32	9%	354
- Medicine (n, %)	51	18%	211	74%	8	3%	17	6%	287
- Pharmacy (n, %)	24	29%	54	65%	2	2%	3	4%	83
- Physiotherapy (n, %)	10	15%	43	66%	3	5%	9	14%	65
- Midwifery (n, %)	8	24%	24	71%	1	3%	1	3%	34
Students per institution (median, [IQR])	6 [4; 7]		8 [6; 10]		6 [4; 6]		10 [7; 12]		8 [4; 10]
Disciplines represented in intervention groups at schools (median, [IQR])	4 [3; 4]		3 [2; 4]		2 [2; 3]		3 [2; 3]		3 [2; 4]

### IPE during training and actions

**Fig. 1:**
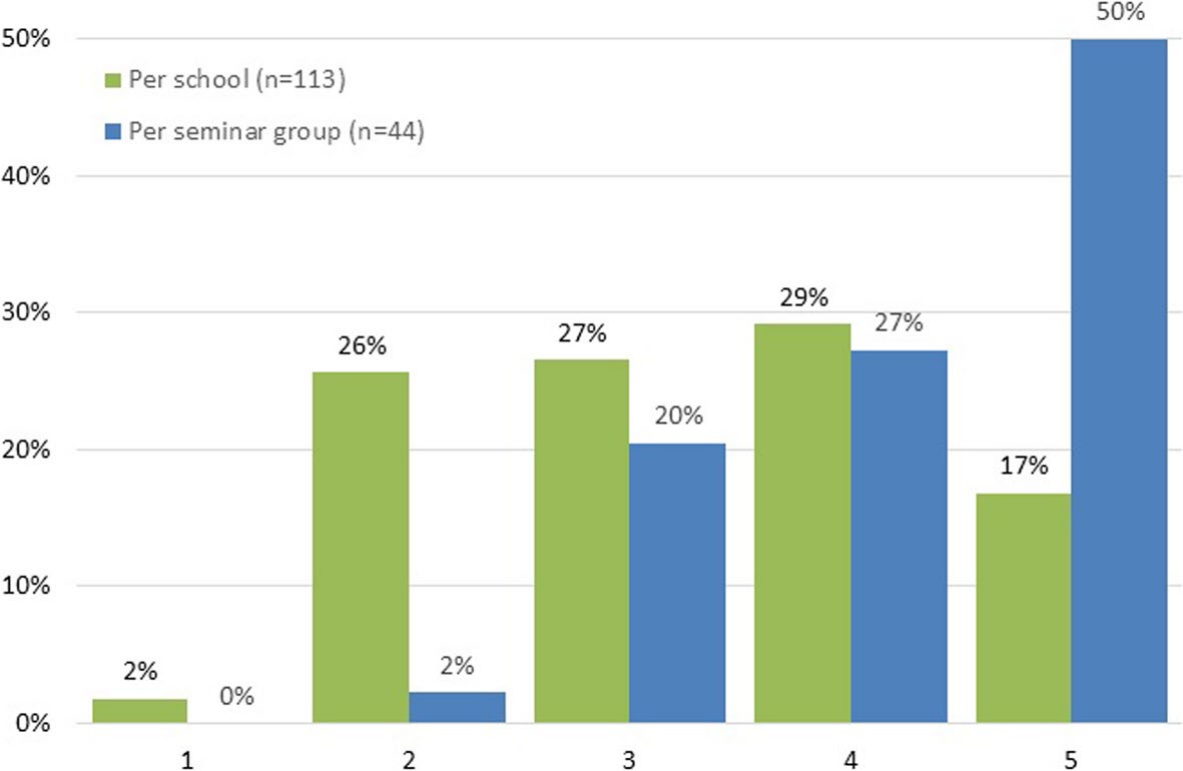
Number of disciplines represented in intervention groups at schools and in seminar groups

The distribution of students across seminar groups, with each group comprising 16 to 20 students, revealed that half of the seminars included at least one student from each of the five undergraduate training programs. However, one seminar consisted of students from only two disciplines, as shown in Fig. 1. In the intervention groups, which ranged from 2 to 10 students per group, the distribution of students' major training program affiliations was more varied. The median number of different program affiliations per group was 3, with an interquartile range of [2;4].

### Students' perception of IPE

In the training seminar, a vast majority of students (75%) recognized the value of Interprofessional Education (IPE), stating that it enhanced the seminar's relevance and appeal, and promoted collaborative learning, as indicated in Table 2. Additionally, a strong majority of students (70%) appreciated the interdisciplinary nature of the interventions, favoring collaborative practice. Despite this, 86% of them felt that IPE complicated the planning of interventions.

**Table 2:** Students' experience and attitudes towards Interprofessional Education (IPE) in the PHS

	Positive Response Rate (%) by Field of Study						*p*
	Nursing	Medicine	Physiotherapy	Pharmacy	Midwifery	**Total**	
**IPE during the seminar**							
Interest in IPE learning	75	73	87	58	88	**75**	0.06
IPE contributing to the relevance of the seminar	64	69	81	50	92	**68**	0.01
IPE enhancing the interest of the seminar	66	68	74	50	88	**68**	0.06
IPE promoting collective learning	63	58	61	58	76	**62**	0.57
**IPE in the intervention**							
Interest in conducting interventions with IPE	69	65	84	69	84	**70**	0.16
Collaborative practice enhanced by IPE	67	61	74	65	88	**67**	0.12
Increased efficiency due to IPE	52	41	52	38	56	**48**	0.28
Intervention planning made easier through IPE	15	15	03	12	12	**14**	0.46
Variations in academic year levels among students enhancing intervention quality	55	54	61	23	68	**54**	0.01
**IPE in the entire PHS**							
IPE allowing the discovery of other disciplines	43	41	32	19	52	**40**	0.11
IPE enabling better understanding of other disciplines	50	48	52	35	64	**49**	0.34
IPE promoting experience of the collaborative nature of future professional activity	62	57	55	50	57	**60**	0.08

Although 60% of students felt that IPE in the PHS could promote collaboration in future professional activity, fewer of them discovered or became more familiar with other disciplines (40%).

### Comments by students

In response to an open-ended question seeking feedback on the PHS program as a whole, 265 students provided comments. Out of these, 69 were specifically pertinent to Interprofessional Education (IPE) and were subjected to a detailed thematic analysis, as presented in Table 3 (the full verbatim can be found in the Supplementary material 2). These comments cast IPE as a cornerstone of the PHS curriculum, with students frequently acknowledging its significance and possible indispensability. They used phrases such as 'PHS is crucial for collaboration among health professionals' and 'IPE offers enriching encounters with other health professionals, which is instructive.' Additionally, students recognized that IPE fosters an understanding of teamwork, which is vital in their impending professional roles.

**Table 3:** Thematic analysis of interprofessional feedback

Category	N	(%)	Key words (occurrence)	examples	Training program (N)
Positive IPE Experience	45	(56)	Interesting (13), Collaboration (12), Exchanges (5), Work (5), Enriching (4), Important (3), Relationships (3), Very good (3), Contribution (2), Knowledge (2), Contact (2), Discovery (2), Essential (2), Profession (2), Fundamental (2), Common project (2), Meetings (2)	“PHS is important for collaborating among health professionals, to get to know the other, and to foster exchanges with future colleagues and professional relationships." "I loved meeting other health professionals and working with them was very instructive." “PHS was a good experience to learn to work with other sectors: something we will each have to do in our respective practices."	Nursing (18) Medicine (15) Physiotherapy (5) Pharmacy (3) Midwifery (4)
IPE Organizational Difficulties	25	(31)	Complexity (2), Organization (8), Planning (8), Disparities (4), Time-consuming (3), Constraints (3), Difficulties (3)	"Interdisciplinarity was complex to manage throughout the project, given the investments and the disparate schedules among disciplines and individuals." "It is difficult to organize between sectors because each one has obligations (internships, exams) which makes the distribution of work quite inequitable."	Nursing (9) Medicine (6) Physiotherapy (4) Pharmacy (3) Midwifery (3)
Negative IPE Experience	11	(14)	Maturity differences (2), Of little use (2), Disparities (2)	"We are completely out of sync with other sectors that do it in the 2nd or 3rd year, particularly in terms of maturity and experience." "I was very disappointed with how some sectors behaved with others because of certain prejudices."	Nursing (6) Medicine (4) Physiotherapy (0) Pharmacy (1) Midwifery (0)

Notwithstanding this positive stance, students also reported substantial challenges associated with IPE, particularly when it came to organizing collaborative efforts. These challenges were predominantly ascribed to the incompatibility of academic schedules between different undergraduate programs. Expressions like 'Interdisciplinarity was complex to manage' illustrate these logistical issues. A comment encapsulating this sentiment was: 'While interdisciplinary work is rewarding, coordinating interventions amidst conflicting academic and internship schedules across disciplines is challenging.'

Furthermore, there were candid reflections on negative experiences within IPE, where issues such as 'maturity differences' and the perceived 'futility' of some activities were cited. Comments like 'We are completely out of sync with other sectors…' and disappointments stemming from 'prejudices' between different sectors underscore the multifaceted nature of student experiences with IPE.

## Discussion

Our study on the PHS program demonstrated a significant commitment and a generally positive perception of IPE, especially during seminars and preventive interventions in schools. However, there were more nuanced views regarding project development, highlighting difficulties related to common organization in interprofessional settings.

The generally positive experience reported in this study aligns with the literature [19–21]. Students emphasized that the IPE experience was the most interesting and enriching aspect of this action-oriented training program. These are encouraging results, showing that undergraduate students appreciate working with other health-related disciplines, despite a lack of common knowledge. It is crucial to note that this training program is the sole initiative of its kind for the five concerned undergraduate training programs at our university level. Although the value of IPE is widely recognized in the pedagogical scientific literature, it remains marginal in many French universities [5, 22–24]. Our experience has shown that it is feasible to synchronize schedules and enable collaboration among students from five distinct undergraduate programs on complex primary prevention projects. This is significant, especially considering the challenges of integrating various health disciplines into a cohesive training program [25, 26]. This challenge is evident in the experiences of other French universities, where PHSs are implemented without integrating the five undergraduate programs [14, 27, 28]. While such pedagogical projects are challenging to implement in terms of resources (shared secretariat, steering committee, pedagogical meetings of undergraduate training program leaders), the positive student experience, coupled with the reality of implemented IPE, is encouraging and could inspire the establishment of other practical teaching programs in French universities (practical clinical work, simulation exercises, care projects in clinical services) [6, 8, 14, 29, 30].

While the experiences were generally positive, our study nonetheless objectified real problems generated by the interprofessional organization of this program. While these issues do not seem to be of a nature to question the general interest of this teaching, they must be taken into account in view of improving the student experience and pedagogical effectiveness. Some of the problems raised seem inherent to interprofessionalism. Numerous studies have shown that students working interprofessionally face communication problems, and that misunderstandings within groups occur. Especially in undergraduate training, it is logical to note a lack of knowledge and openness towards other health training programs. These problems are encountered in professional practice, and it is interesting that students find themselves confronted early in their studies with such situations, in order to better understand and deal with them in their future professional practice [4, 5, 22–24, 31].

If problems inherent to IPE have been found, characteristics specific to our training program may explain some of the mixed experiences and attitudes of our students. The first issue concerns the different years of study participating in the PHS. Indeed, medical and physiotherapy students are in their 3rd year, nursing and midwifery students in their 2nd year, while pharmacy students are in their 5th year. These differences result in different levels of maturity among the students, in terms of both age and advancement in their studies. These differences are likely to exacerbate the heterogeneity between the disciplines involved in the program and may lead to further misunderstandings and ignorance among students at different levels. It is regrettable that when the PHS was established at the nationwide level, each national undergraduate training program authority chose a year without overall consultation and harmonization. This option characterizes an operation that is too compartmentalized, similar to what is seen in the tradition of health education [32, 33]. Our study could be useful for the authorities, as a way if encouraging for global reflection on the harmonization of the years of study included in the PHS.

Another explanation concerns the harmonization of the schedule, which freed up all Monday afternoons over three months. Our results showed that appointments between students, as well as appointments with schools for prevention interventions, were difficult to coordinate, with some students being unavailable. These difficulties show that, even by allocating dedicated time for interprofessional work, students' schedules can be impacted by burdens specific to each undergraduate training program calendar. It is indeed conceivable that students with a clinical internship in the morning, or an exam the next day, may be less available than students without these burdens. For instance, it can be specified here that 5th-year pharmacy students in France have many exams sanctioning their future professional practice, given that for them, the year will culminate in a nationwide competition. Although this explanation is only hypothetical, it could partially explain why students from this undergraduate training programs generally have a less positive experience than others. Above and beyond the harmonization of the years of study included in the PHS, it may be necessary to identify more significant common time slots, to lighten other workloads, consequently allowing better participation of all students, regardless of their undergraduate training programs.

## Study limitations

The present study, while providing important insights into the PHS program, has several limitations. The first and foremost is our decision not to use the Readiness for Interprofessional Learning Scale (RIPLS), an established tool in medical pedagogy [34]. Instead, we opted for a bespoke questionnaire specifically designed to capture the unique experiences and attitudes of students in the PHS program, which focuses on primary prevention interventions in schools [13, 17, 18]. This approach, aimed at addressing the specificities of the PHS context, limits the comparability of our findings with other studies utilizing the RIPLS. In future research, incorporating such standardized instruments could allow for more extensive comparisons and a more thorough assessment of IPE initiatives.

Moreover, the response rate of 42% for our survey, though aligning with typical online survey responses, might not comprehensively represent the entire range of student experiences and perceptions. The study's primary reliance on quantitative data, augmented by qualitative feedback, indicates that more in-depth qualitative methods such as interviews or focus groups could provide richer, more detailed insights [5]. Another limitation is the potential for selection bias, as students with strong opinions (either positive or negative) might have been more inclined to participate.

## Conclusion

This study on the PHS program highlights its effectiveness in promoting IPE among healthcare students. Key findings indicate high student satisfaction with practical teaching sessions and primary prevention projects in schools, underscoring the value of hands-on IPE experiences. However, challenges in scheduling and coordination across disciplines were notable concerns. Feedback suggests that while the PHS program is beneficial for fostering collaborative practice, it requires further refinement, particularly in organizational aspects. These insights elucidate IPE and underscore the need for continuous development of IPE program structures addressed to future healthcare professionals.

### Supplementary Information

**Supplementary Material 1. **

**Supplementary Material 2. **

## Data Availability

The datasets generated and/or analysed during the current study are available from the corresponding author on reasonable request.
