# Supplementary material for: Insights of undergraduate health sciences students about a French interprofessional training initiative
Source: BMC Med Educ. 2024 Mar 1;24:220. doi: 10.1186/s12909-024-05212-9 (PMC10908004; doi:10.1186/s12909-024-05212-9)
Supplement: Supplementary file 1 — Supplementary Material 1. [file 12909_2024_5212_MOESM1_ESM.docx]

Supplementary material 1: Student’s intervention reports analysis

**Supplemental table: Student Work (n=108 institutions)**

|  | Kindergarten  n=23 | Elementary  n=70 | Middle School  n=6 | High School  n=9 | Total  n=108 | P |
| --- | --- | --- | --- | --- | --- | --- |
| Number of Sessions | 5 [5 ; 5] | 5 [5 ; 5] | 5 [5 ; 8] | 4 [3 ; 5] | 5 [5 ; 5] | <0,01 |
| Duration of Sessions, h | 1 [1 ; 1] | 1 [1 ; 1] | 1 [1 ; 1] | 1 [1 ; 1] | 1 [1 ; 1] | 0,98 |
| Student Work Time | 19 [14 ; 27] | 20 [17 ; 27] | 28 [28 ; 39] | 28 [20 ; 34] | 21 [17 ; 28] | 0,01 |
| Including travel time | 4 [3 ; 6] | 4 [3 ; 5] | 6 [3 ; 9,5] | 5 [3 ; 8] | 5 [5 ; 6] | 0,07 |
| Including preparation time | 8 [6 ; 11] | 11 [8 ; 17] | 16 [12 ; 18] | 15 [10 ; 21] | 10 [8 ; 16] | 0,03 |
| Including action time | 5 [4 ; 5] | 5 [5 ; 6] | 5 [5 ; 10] | 6 (4 ; 7] | 5 [5 ; 5] | 0,90 |

Number of Sessions, Duration and Time are expressed using (median, [IQR])
