# Supplementary material for: Insights of undergraduate health sciences students about a French interprofessional training initiative
Source: BMC Med Educ. 2024 Mar 1;24:220. doi: 10.1186/s12909-024-05212-9 (PMC10908004; doi:10.1186/s12909-024-05212-9)
Supplement: Supplementary file 2 — Supplementary Material 2. [file 12909_2024_5212_MOESM2_ESM.docx]

Supplementary material 2: Full verbatim of the 69 free-text comments related to IPE experience

| ID | Verbatim | Categories | keywords | training program |
| --- | --- | --- | --- | --- |
| 1 | Interdisciplinary work was complex to manage throughout the project, given the investments and disparate schedules among disciplines and individuals | IPE Organizational Difficulties | Complex / Time Disparities between Disciplines / Schedules | Physiotherapy |
| 2 | A good exercise in collaboration and organization in interdisciplinarity | Positive IPE Experience | Collaboration / Organization | Nursing |
| 3 | Interdisciplinarity of little use but almost impossible to organize with the timetables according to the sectors (some in internships, some had exams, or simply different available slots according to the courses) | Negative IPE Experience / IPE Organizational Difficulties | Of Little Use / Complex / Planning | Nursing |
| 4 | Thank you for allowing us to carry out these missions with other health sectors, it was much more enriching on all levels for everyone | Positive IPE Experience | Enriching / Missions | Physiotherapy |
| 5 | The exercise in interdisciplinarity is one of the most relevant aspects of the PHS | Positive IPE Experience | Relevant | Medicine |
| 6 | It is difficult to organize between sectors because everyone has obligations (internships, exams...) which makes the distribution of work quite inequitable | IPE Organizational Difficulties | Difficulties / Organization / Time Disparities between Disciplines / Inequitable | Physiotherapy |
| 7 | Interdisciplinarity is a plus | Positive IPE Experience | Plus | Nursing |
| 8 | Organize schedules to have the same course hours to optimize interdisciplinarity | IPE Organizational Difficulties | Schedules / Optimize | Nursing |
| 9 | However, the administrative forcing of this interdisciplinarity imposes significant organizational constraints for students from different sectors who have different schedules | IPE Organizational Difficulties | Schedules / Constraints | Medicine |
| 10 | Interdisciplinarity allowed me to discover and understand other sectors and to exchange with students | Positive IPE Experience | Discovery / Understanding / Exchanges | Nursing |
| 11 | The concept of interdisciplinarity has always been essential for me | Positive IPE Experience | Essential | Nursing |
| 12 | We are totally out of sync with other sectors that do it in the 2nd or 3rd year, particularly in terms of maturity and experience | Negative IPE Experience / IPE Organizational Difficulties | Maturity / Misalignment | Pharmacy |
| 13 | PHS important for collaborating between health professionals, to know the other, and to foster exchanges with future colleagues and professional relationships | Positive IPE Experience | Collaborate / Exchange / Future Colleagues / Relationships | Nursing |
| 14 | For me, interdisciplinarity is crucial in our practice, and the health service is a very enriching first experience | Positive IPE Experience | Crucial / Profession / Enriching | Physiotherapy |
| 15 | The organization to create interventions remains complicated given the different schedules of each sector | IPE Organizational Difficulties | Complex / Schedules | Midwifery |
| 16 | It was a good experience thanks to interdisciplinarity | Positive IPE Experience | Good | Midwifery |
| 17 | Good experience in multi-professionalism | Positive IPE Experience | Good | Nursing |
| 18 | Very interesting, a very good interdisciplinary experience that brings a lot to students and the targeted public | Positive IPE Experience | Interesting / Contribution | Midwifery |
| 19 | Essential to work in interdisciplinarity | Positive IPE Experience | Essential | Medicine |
| 20 | Organizational difficulties in interdisciplinarity but interesting exchanges | Positive IPE Experience / IPE Organizational Difficulties | Difficulties / Organization / Exchanges / Interesting | Midwifery |
| 21 | Very good overall experience, interdisciplinarity is very interesting if everyone puts in their effort | Positive IPE Experience | Interesting / Collaboration | Medicine |
| 22 | The relational aspects and bridges between different professions are increasingly essential in current issues | Positive IPE Experience | Essential / Relationships | Medicine |
| 23 | Very enriching seminar, it is necessary for me to have more interprofessional approach in our studies! | Positive IPE Experience | Enriching / Need for More IPE in Studies | Pharmacy |
| 24 | Multidisciplinarity is great as long as everyone is involved | Positive IPE Experience / IPE Organizational Difficulties | Great / Involvement | Nursing |
| 25 | Concordance of schedules in interdisciplinarity | IPE Organizational Difficulties | Schedules | Midwifery |
| 26 | Interdisciplinary work is interesting but not under these conditions: the difference in the progress of studies between sectors (1st or 2nd year in nursing studies vs 5th year in pharmacy) makes collaboration difficult due to a difference in maturity | Positive IPE Experience / IPE Organizational Difficulties | Interesting / Maturity / Collaboration / Difficult | Pharmacy |
| 27 | Interdisciplinarity brings a real renewal to health training | Positive IPE Experience | Renewal | Nursing |
| 28 | Trying at all costs to impose interdisciplinarity while, in fact, it complicates the organization of the health service without perceptible benefits | Negative IPE Experience / IPE Organizational Difficulties | Impose / Complex / Organization / Of Little Use | Medicine |
| 29 | Lack of interdisciplinarity due to geographical practicality | Negative IPE Experience | Lack / Geographical Distance | Medicine |
| 30 | Some sectors less invested | Negative IPE Experience | Disparities | Medicine |
| 31 | The relationship with medical students was very complicated | Negative IPE Experience | Relationship / Complex | Nursing |
| 32 | Very good experience but takes a lot of time in addition to other exams and requires significant investment | Positive IPE Experience / IPE Organizational Difficulties | Very Good / Time-Consuming / Significant | Nursing |
| 33 | It's good to be forced to be with people outside of medicine even if it makes organization more complicated | Positive IPE Experience / IPE Organizational Difficulties | Good / Constraints / Complex | Medicine |
| 34 | The interest of this seminar is to get to know other students | Positive IPE Experience | Knowledge / Interest | Medicine |
| 35 | Interdisciplinarity is sometimes complicated in terms of organization | IPE Organizational Difficulties | Complex / Organization | Pharmacy |
| 36 | Interdisciplinarity makes organization very difficult | IPE Organizational Difficulties | Complex / Organization | Medicine |
| 37 | Allowed the creation of cohesion in a multi-professional group | Positive IPE Experience | Cohesion | Nursing |
| 38 | Useful but not necessarily in interdisciplinarity | Negative IPE Experience | Not Useful | Nursing |
| 39 | I loved meeting other health professionals and working with them was very instructive | Positive IPE Experience | Meetings / Instructive | Medicine |
| 40 | Planning between sectors does not match, in addition to our internships during the intervention dates at the prevention site | IPE Organizational Difficulties | Schedules | Nursing |
| 41 | Interesting experiences but frustrated by not having had more time to dedicate to it | Positive IPE Experience / IPE Organizational Difficulties | Interesting / Time-Consuming | Nursing |
| 42 | I am not sure that it promotes future collaboration between sectors | Negative IPE Experience | Uncertain / Collaboration | Nursing |
| 43 | Interdisciplinary complicates things for organization | IPE Organizational Difficulties | Complex / Organization | Physiotherapy |
| 44 | Understanding the importance of collaboration in our professions | Positive IPE Experience | Important / Profession / Collaboration | Nursing |
| 45 | For a more effective interdisciplinary collaboration, more diversity in the group is needed | IPE Organizational Difficulties | Diversity / Collaboration | Nursing |
| 46 | The health service is interesting to strengthen collaboration work | Positive IPE Experience | Interesting / Strengthen / Work / Collaboration | Midwifery |
| 47 | For interdisciplinarity, there is an educational interest | Positive IPE Experience | Educational Interest | Medicine |
| 48 | Interdisciplinary work is an enriching experience, the only problem is that it is difficult for all sectors to organize interventions with stages and other school imperatives | Positive IPE Experience / IPE Organizational Difficulties | Enriching / Complex / Organization / Constraints | Physiotherapy |
| 49 | The PHS was a good experience to learn to work with other sectors: this is what we will each have to do in our respective practices | Positive IPE Experience | Learn / Practices | Medicine |
| 50 | Good to work in multi-professionalism | Positive IPE Experience | Good / Work in Multi-Professionalism | Nursing |
| 51 | Interdisciplinarity brought nothing to the seminar | Negative IPE Experience | No Contribution | Nursing |
| 52 | It's interesting to discover students from other sectors, to be able to exchange and build a common project | Positive IPE Experience | Interesting / Discovery / Exchange / Building / Common Project | Physiotherapy |
| 53 | Multidisciplinarity also allowed making new acquaintances and each bringing complementary ideas to the project | Positive IPE Experience | Acquaintances / Complementary / Project | Medicine |
| 54 | Interdisciplinarity and health promotion among the young are very important for our future profession | Positive IPE Experience | Important / Future Profession / Health Promotion | Medicine |
| 55 | Interesting, allows a first contact with multidisciplinarity | Positive IPE Experience | Contact / Interesting | Nursing |
| 56 | It was very interesting to be in interdisciplinarity. | Positive IPE Experience | Interesting | Nursing |
| 57 | Interdisciplinarity is sometimes complicated due to different internship hours. | IPE Organizational Difficulties | Complex/ Schedules | Medicine |
| 58 | Interdisciplinarity complicated when half of the participants do not participate. | Negative IPE Experience / IPE Organizational Difficulties | Complex/ Participation Disparity | Nursing |
| 59 | Excellent training that allows contact with all health students. | Positive IPE Experience | Excellent/ Contacts | Medicine |
| 60 | Meeting other students from different disciplines is a contribution. | Positive IPE Experience | Meeting/ Contribution | Nursing |
| 61 | I was very disappointed with how some disciplines behaved with others due to certain prejudices. | Negative IPE Experience | Disparities, Prejudices | Medicine |
| 62 | Good experience exchanging with colleagues from different specialties. | Positive IPE Experience | Exchanges/ Colleagues | Medicine |
| 63 | The most interesting thing is working in interdisciplinarity. | Positive IPE Experience | Interesting/ Work | Medicine |
| 64 | Interdisciplinarity has allowed bringing different viewpoints and knowledge. | Positive IPE Experience | Different Knowledge/ Different Viewpoints | Nursing |
| 65 | Inter-sector project very interesting but quite time-consuming. | Positive IPE Experience / IPE Organizational Difficulties | Interesting/ Time-Consuming | Medicine |
| 66 | There are always sectors that have less time than others. | IPE Organizational Difficulties | Time Disparities Between Disciplines | Nursing |
| 67 | Very good to work with students from other sectors. | Positive IPE Experience | Very Good/ Work | Physiotherapy |
| 68 | I appreciated this work in multi-professionalism. | Positive IPE Experience | Appreciate | Nursing |
| 69 | Very good to work with other sectors. | Positive IPE Experience | Very Good/ Work | Pharmacy |
